# Optimum Contact Configurations for Quasi-One-Dimensional Phosphorene Nanodevices

**DOI:** 10.3390/nano13111759

**Published:** 2023-05-29

**Authors:** Mirko Poljak, Mislav Matić

**Affiliations:** Computational Nanoelectronics Group, Faculty of Electrical Engineering and Computing, University of Zagreb, 10000 Zagreb, Croatia; mislav.matic@fer.hr

**Keywords:** phosphorene, black phosphorus, nanoribbon, quasi-one-dimensional, quantum transport, transfer length, contact resistance, non-equilibrium Green’s function (NEGF) formalism

## Abstract

We employ atomistic quantum transport simulations based on non-equilibrium Green’s function (NEGF) formalism of quasi-one-dimensional (quasi-1D) phosphorene, or phosphorene nanoribbons (PNRs), to explore routes towards minimizing contact resistance (*R_C_*) in devices based on such nanostructures. The impact of PNR width scaling from ~5.5 nm down to ~0.5 nm, different hybrid edge-and-top metal contact configurations, and various metal–channel interaction strengths on the transfer length and *R_C_* is studied in detail. We demonstrate that optimum metals and top-contact lengths exist and depend on PNR width, which is a consequence of resonant transport and broadening effects. We find that moderately interacting metals and nearly edge contacts are optimum only for wider PNRs and phosphorene, providing a minimum *R_C_* of ~280 Ωμm. Surprisingly, ultra-narrow PNRs benefit from weakly interacting metals combined with long top contacts that lead to an added *R_C_* of only ~2 Ωμm in the 0.49 nm wide quasi-1D phosphorene nanodevice.

## 1. Introduction

Since the discovery of graphene [1], tremendous research efforts have been invested into exploring the properties and technological applicability of novel two-dimensional (2D) or van der Waals materials [2]. The 2D materials cover various material classes, from semiconductors to dielectrics [3,4,5], which facilitates the development of electron devices such as field-effect transistors (FETs) with atomically thin channels [6,7]. A recent ab initio study has predicted more than 4000 2D materials to be stable under normal conditions, with exfoliation foreseen to be possible for several hundred from their bulk counterparts [8,9]. The ultimate thickness, dangling bond-free surfaces, and good electronic and transport properties make many 2D semiconductors promising candidates to replace nanoscale silicon FETs as the fundamental building block in the semiconductor electronics industry. Monolayer black phosphorus (BP), or phosphorene [10,11,12], is among such promising 2D semiconductors due to its acceptable bandgap, adjustable between ~0.3 eV and ~2 eV depending on the number of monolayers in BP, and adequate transport properties [13,14]. For the latter, high carrier mobilities of ~1.000 cm^2^/Vs were measured in BP FETs [15], but only in relatively thick samples. The mobility in phosphorene was discussed in detail in [16], where disappointingly low mobilities of 25 cm^2^/Vs at 300 K are calculated by combining ab initio and Monte Carlo simulations. Therefore, any phosphorene FETs relevant to technology applications necessarily need to be short-channel devices in which the contribution of ballistic transport is expected to dominate the device performance [17].

In addition to thickness dependence, the properties of phosphorene can be further modulated by quantum confinement effects. Namely, reducing the dimensionality of phosphorene could lead to new physical properties that could improve device characteristics. Patterning phosphorene into quasi-one-dimensional (quasi-1D) structures, such as phosphorene nanoribbons (PNRs), has been experimentally demonstrated recently [18,19] where PNR widths under ~4 nm and bandgaps up to ~1.8 eV have been reported. Hence, it is now possible to imagine realistically sized PNR-based FETs [20,21] that could enable high integration densities on-chip, combining atomically thin channels and ideal gate-control over the electrostatics with promising carrier transport. While the performance of phosphorene and PNR FETs with either ballistic or diffusive transport have been investigated previously by numerical simulations at the scaling limit [21,22,23], very little is known about the properties of contact resistance (*R_C_*) in such nanostructures [24,25,26,27], which is the main technology limiter for large-area 2D materials. The *R_C_* reported in multilayer BP [28,29,30] is at least one order of magnitude higher than the elusive quantum limit of contact resistance of ~30 Ωµm [27,31], and unacceptable for future logic device technology generations where *R_C_* < 135 Ωµm is needed according to the International Roadmap for Devices and Systems (IRDS) [32]. Contact resistance masks promising transport properties, so it is imperative to understand *R_C_* behavior not only for nanoscale FETs but also for other applications where the information is carried by the electron current.

In this paper, we investigate *R_C_* behavior in quasi-1D phosphorene nanostructures by employing atomistic quantum transport simulations for various PNR sizes and contact configurations. The impact of PNR width scaling, area scaling in hybrid edge-and-top contacts, and of varying metal–channel interaction strengths on the transfer length and *R_C_* is reported and discussed in detail. We find that wider PNRs benefit from moderately interacting metals with short top contacts, whereas longer top contacts and weakly interacting metals are needed for extremely narrow PNRs to achieve minimum *R_C_* close to the quantum limit.

## 2. Methods

**Figure 1:**
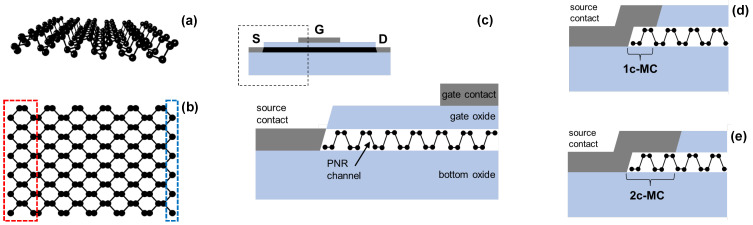
Illustration of a phosphorene nanoribbon with armchair edges with a side view shown in (**a**) and top view in (**b**). (**c**) Schematic view of the simulated PNR FET with MCs, illustrating the edge contacts labeled as 1a-MCs in the bottom figure. Illustration of two hybrid edge-and-top MC configurations showing (**d**) 1c-MCs and (**e**) 2c-MCs where metal electrodes interact with the first and the first two super-cells, respectively.

The equilibrium part of the NEGF formalism is employed to obtain relevant device transport and electronic properties. Our in-house NEGF code [33,34,35] is used in the simulations that solve Schrödinger’s equation for the device with open boundary conditions towards two electrodes, i.e., source/drain (S/D) contacts. The retarded Green’s function of the device takes as input the device Hamiltonian based on the tight-binding (TB) model from [36] that was fitted on ab initio band structure. The second and third inputs are the contact self-energy matrices, Σ*_S_^R^* and Σ*_D_^R^*, for the source and drain contact, respectively. Figure 1a,b illustrate a semiconducting armchair PNR where the dashed-line rectangle on the left side of Figure 1b marks a super-cell along the nanoribbon width (*W*), which is repeated along the length (*L*) to construct the total Hamiltonian matrix. The retarded and advanced Green’s functions are then utilized to find the transmission function of the PNR and its density of states (DOS). The PNR FETs are self-consistently simulated using the top-of-the-barrier (TOB) model that provides ballistic device characteristics based on full band structure in the channel and S/D regions. Channel width varies from ~0.5 nm to ~5.5nm, while the length is set to 15 nm for all devices because the explored design space is vast as will be shown later. Moreover, 15 nm long channels are of interest for future CMOS technology nodes as stated in the IRDS [32]. All devices have the same supply voltage (0.7 V), equivalent oxide thickness (1 nm), doping (0.001 molar fraction of the atomic areal density), and threshold voltage (~0.24 V) [35], for a meaningful comparison of PNR FETs with different nanoribbon/channel widths and contact configurations.

Contact resistance is calculated from the difference in the drain current between the PNR FET with ideal contacts (IC) and their counterparts with metal contacts (MC) [37]. The ICs are treated with the standard approach, i.e., fast iterative Sancho–Rubio method [38], and devices with ICs exhibit perfect step-like transmission characteristics and DOS curves with van Hove singularities [39,40]. In contrast, MCs are implemented with a wide-band limit (WBL) model [39] where the metal–nanoribbon interaction strength is set to a constant complex value, which is equivalent to having constant hopping between metal and channel atoms and a constant metal DOS at Fermi level. It has been reported previously that the WBL model is adequate for the study of metallization effects and *R_C_* in nanostructured devices, including those based on 2D and quasi-1D materials [26,41,42]. Metal–phosphorene interfaces have been investigated previously by ab initio methods [43,44,45], but due to high computational complexity, these studies are limited to electronic properties, with little consideration of carrier transport in realistically sized devices. In contrast, our approach enables quantum transport simulations of phosphorene nanostructures consisting of thousands of atoms. Within the WBL model, the metal–nanoribbon interaction strength is assumed to be equal for S/D contacts and is designated as −ImΣ*_S_^R^* = −ImΣ*_D_^R^* = −ImΣ. The −ImΣ varies by orders of magnitude depending on the MC material, e.g., from ~0.01 eV to ~20 eV in carbon nanotubes [41,46]. Therefore, we start with the initial −ImΣ of 0.9 eV and afterward use different −ImΣ values to study the impact of various metals on *R_C_* in quasi-1D phosphorene.

This work focuses on studying the impact of different MC configurations, i.e., distinct combinations of edge and top contacts, on the MC-induced added *R_C_* in ultrascaled PNRs at 300 K. Several cases are analyzed with a different number of atom lines or super-cells, counting from the left or right edges of the PNR, with which the S/D metal contacts interact. Pure edge contacts where MCs interact only with edge phosphorus atoms or the first atom line, shown bounded by a rectangle on the right side of Figure 1b, are labeled 1a-MCs and are illustrated in Figure 1c. A similar description is valid for 2a-MCs as well, while the remaining MC configurations are hybrid contacts in which the MCs also extend on top of the PNR by a certain length measured in super-cell widths. For example, 1c-MCs cover one super-cell (Figure 1d), while 2c-MCs extend over two super-cells from the left and right edges of the PNR (Figure 1e). This study identifies optimum MC configurations for the minimum intrinsic *R_C_* in PNRs, depending on device size and electrode material choice.

## 3. Results and Discussion

**Figure 2:**
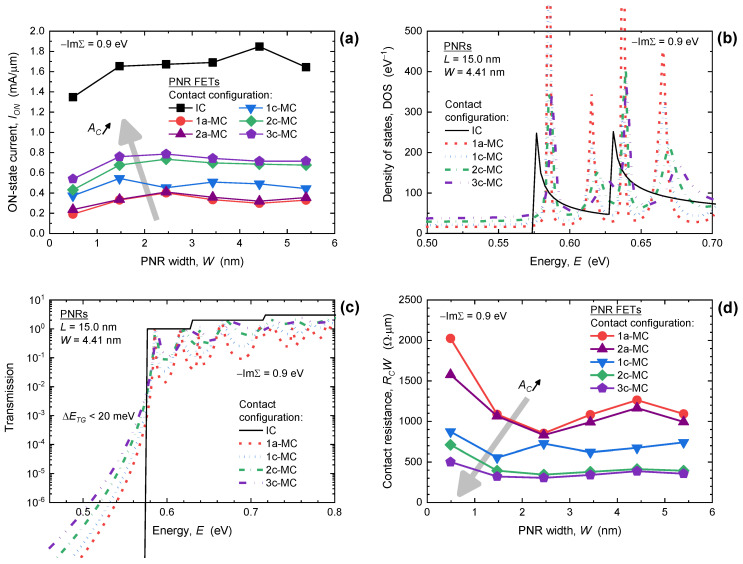
(**a**) Impact of *W*-scaling on *I_ON_* in PNR FETs with ICs and different configurations of MCs. (**b**) DOS and (**c**) transmission of the 4.41 nm wide and 15 nm long PNRs with ICs and MCs. (**d**) Dependence of *R_C_* in PNR FETs on nanoribbon width and contact configuration. In all plots −ImΣ = 0.9 eV.

The impact of width downscaling on *I_ON_* is shown in Figure 2a, and we clearly note a weak *W*-dependence and a strong influence of MC configuration when −ImΣ = 0.9 eV. The *I_ON_* in PNR FETs with ideal contacts slightly decreases in the examined width range, from 1.64 mA/μm to 1.35 mA/μm, whereas all devices with MCs exhibit significantly poorer current-driving capabilities, indicating high *R_C_*. For purely edge MCs (1a-MC case) the *I_ON_* equals 0.33 mA/μm for *W* = 5.40 nm and decreases to 0.19 mA/μm in the 0.49 nm wide PNR FET. When the contact area (*A_C_*) increases from the 1a-MC to the 3c-MC case the ON-state drain current also increases up to 0.72 mA/μm in the widest device. However, the enhancement effect caused by larger *A_C_* saturates in PNR FETs with wider nanoribbon channels. Therefore, it seems that MCs with a larger top part are generally more favorable for ultrascaled phosphorene nanodevices in terms of the drive current.

The origins of *I_ON_* decrease in PNR FETs with MCs in comparison to devices with ICs is elaborated in Figure 2b,c that report DOS and transmission, respectively, in 4.41 nm wide devices with different MC configurations. When metal electrodes are attached to the PNR, the DOS loses the van Hove singularities that are indicative of quasi-1D structures, and Lorentzians are formed away from subband minima due to MC-induced broadening effects [24,26,27]. Decreasing the contact area from the 3c-MC to the 1a-MC case decreases the DOS, which in turn decreases channel charge density that feeds the drain current of a PNR FET. At the same time, metal-induced gap states (MIGS) are induced by MCs, and their intensity inside the bandgap increases as the contact area increases. Nevertheless, the MIGS are strongly localized at the edges of the nanoribbon in the vicinity of contacts and do not contribute to transport [26], which is also visible in Figure 2c, which reports a greatly suppressed transmission inside the bandgap. Therefore, a transport gap (*E_TG_*) exists and is only weakly modulated by MCs in 15 nm long devices with −ImΣ = 0.9 eV. For the 4.41 nm wide PNR, the *E_TG_* decreases when going from edge contacts (1a-MCs) towards the 3c-MC configuration, but the change is negligible being lower than 20 meV. Focusing on the conduction band near the minimum, where the majority of electron current in a FET is distributed, we observe that PNR FETs with 1a-MCs exhibit the lowest transmission which agrees with the lowest *I_ON_* in these devices as reported in Figure 2a. Qualitatively identical effects are observed in the narrowest 0.49 nm wide PNR, as shown in Figure S1 in the Supplementary Materials.

The analyzed *I_ON_* behavior, supported by DOS and transmission comparison for different MC configurations translates into *W*-normalized *R_C_* shown in Figure 2d. Generally, *R_C_* increases when PNR width is scaled down irrespective of the contact shape. For edge contacts, i.e., 1a-MC and 2a-MC cases, *R_C_* increases from ~990 Ωμm at *W* = 5.40 nm up to ~2020 Ωμm for *W* = 0.49 nm. In the same width range, *R_C_* is lowest for the 3c-MC configuration with the resistance going from 355 Ωμm in the widest to 501 Ωμm in the narrowest device. In comparison to *R_C_* measured in few-layer BP devices with top Ni, Ti, and Cr contacts in the literature [30], where *R_C_* from ~140 Ωμm to ~800 Ωμm is reported, our results for the widest PNRs with the longest MCs fit within the experimental range. When we consider *R_C_* modulation by contact area, we observe that *R_C_* decreases considerably with *A_C_* increase for all nanoribbon widths when −ImΣ = 0.9 eV. When changing the MC configuration from the 1a-MC to the 3c-MC case, the *R_C_* improvement is 67% at *W* = 5.40nm and 75% for *W* = 0.49 nm. Moreover, the *R_C_*-*W* curve in Figure 2d seems to overlap for wider devices for the two MC cases with the largest top contact area. These findings demonstrate that 3c-MCs are close to ideal for wider PNR FETs, whereas even longer top contacts are expected to further decrease the *R_C_* in devices with the narrowest quasi-1D phosphorene channels.

**Figure 3:**
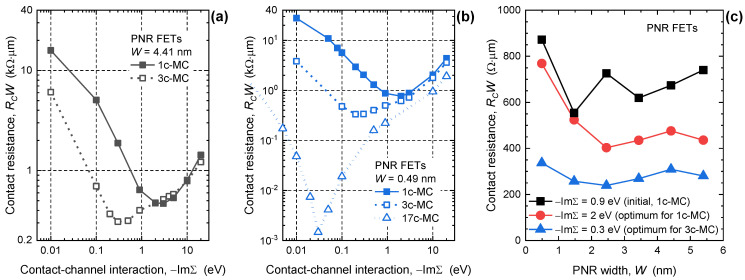
Influence of contact configuration and −ImΣ on *R_C_* of the (**a**) 4.41 nm and (**b**) 0.49 nm wide PNR FET. (**c**) Width dependence of *R_C_* for the chosen configurations with seemingly optimum −ImΣ for 1c- and 3c-MCs.

The findings on *R_C_* are obtained by using −ImΣ = 0.9 eV which represents a moderately interacting metal electrode. On the other hand, various −ImΣ values describing various interaction strengths and metallization effects are possible by employing different metals as shown previously for, e.g., carbon nanotubes [41,46], and as demonstrated qualitatively by ab initio studies of large-area phosphorene–metal interfaces [43,44,47]. Hence, in the following text, we explore the impact of varying contact–channel interaction strength −ImΣ together with the influence of MC configuration on *R_C_* in PNR FETs. Figure 3a,b report the dependence of *R_C_* on −ImΣ and MC shape for the 4.41 nm and 0.49 nm wide device, respectively. The same is reported for *W* = 2.45 nm in Figure S2 in the Supplementary Materials. The characteristic surprisingly exhibits a minimum, which demonstrates the existence of the optimum interaction parameter −ImΣ and, hence, optimum metal electrode materials for PNR FETs. This effect was reported previously in [35] for devices with edge contacts, but here we demonstrate it for hybrid MC configurations that include top contacts as well. As shown in Figure 3a for 4.41 nm wide devices, the optimum −ImΣ is 2 eV and minimum *R_C_* is 468 Ωμm for the 1c-MC case and, hence, the choice of optimum material reduces *R_C_* by 31% in comparison to the data presented in Figure 2d. Moreover, increasing the top contact area by using 3c-MCs pushes the optimum −ImΣ to 0.3 eV and minimum *R_C_* to 309 Ωμm. The same optimum −ImΣ values, i.e., 2 eV and 0.3 eV for the 1c-MC and 3c-MC case, respectively, and similar qualitative behavior of *R_C_* is observed also for the ultimately scaled 0.49 nm wide PNR FET shown in Figure 3b. Hence, optimum metal–PNR interaction strength depends on the channel material and not on the quantum confinement effects in narrow nanoribbons. These findings show that in PNR FETs the hybrid contact configurations with longer top contacts benefit from weakly interacting MCs, which is somewhat counterintuitive. In addition, using strongly interacting metals with −ImΣ > 3 eV results in higher *R_C_* that is only negligibly modulated by top contacts.

In order to assess the benefits of employing optimum MCs, in Figure 3c we plot *R_C_*-*W* characteristics for PNR FETs with 1c-MCs and its optimum −ImΣ = 2 eV, and for PNR FETs with 3c-MCs and its optimum −ImΣ = 0.3 eV. The figure contains the results for the initial 1c-MC device with −ImΣ = 0.9 eV reported in Figure 2d for comparison. For the 1c-MC case, the improvement of *R_C_* is especially evident for wider nanoribbon where *R_C_* decreases from 740 Ωμm to 436 Ωμm, i.e., by 41% for *W* = 5.40 nm. The change is much weaker in narrower devices, with *R_C_* decreasing from 872 Ωμm to 769 Ωμm, i.e., by only 12% in the 0.49 nm wide device. When the top contact area further increases in the 3c-MC case, the *R_C_* is additionally reduced when the optimum −ImΣ = 0.3 eV is used, in comparison to Figure 2d which shows the data obtained with suboptimum MCs. The minimum *R_C_* is only 239 Ωμm for *W* = 2.45 nm and increases with *W*-downscaling to the worst-case value of 337 Ωμm in the 0.49 nm wide PNR FET. Therefore, the choice of MC configuration and metal–channel interaction strength can be adopted to reduce *R_C_* considerably to ~240 Ωμm, but even this *R_C_* value is still considerably larger than the quantum limit of contact resistance in PNRs and other 2D and 1D materials and structures [27]. The *R_C_* can indeed be minimized further, as we will show later in the text, but first, we wish to explain the origin of the optimum metal–PNR interaction strength.

**Figure 4:**
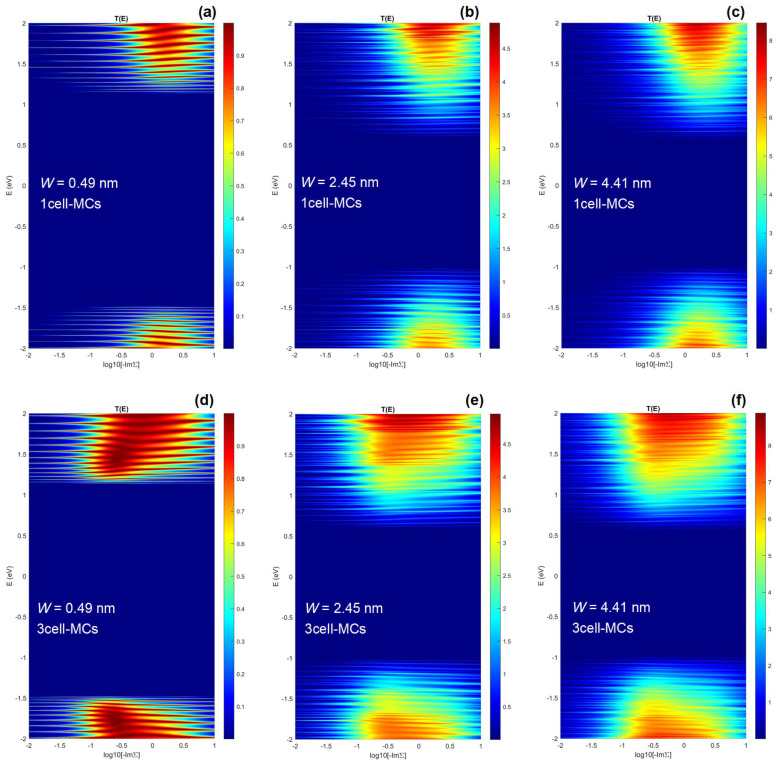
Dependence of the energy-resolved transmission on the metal–channel interaction strength for 0.49 nm, 2.45 nm, and 4.41 nm wide PNRs with (**a**–**c**) 1c-MCs and (**d**–**f**) 3c-MCs.

The root of the optimum −ImΣ and the accompanying minimum intrinsic *R_C_* in quasi-1D structures of 2D materials is made clear by examining the energy and interaction-dependent transmission characteristics. Figure 4 reports the *T*(*E*, −ImΣ) with *x*-axis in the logarithmic scale for 0.49 nm, 2.45 nm, and 4.41 nm wide PNRs with 1c-MCs (top row) and 3c-MCs (bottom row). The calculations are performed for six different device configurations (three PNR widths and two MCs), each with 51 −ImΣ points and 2.000 energy points, resulting in a total of 612.000 NEGF calculations for 306 devices for the data reported in Figure 4. For all devices, there exist the expected optimum energy ranges for transmission, i.e., conduction and valence bands of the nanostructure, and also the unexpected optimum −ImΣ range in which the transmission is high. For low −ImΣ, the transport occurs only via tunneling or hopping, and the transmission is greatly suppressed due to poor coupling between the channel and the two electrodes [41]. Similarly, very large −ImΣ causes greater broadening that merges individual transmission peaks and leads to an overall transmission reduction [26,35]. A clearer insight into the behavior of transmission characteristics is provided for the 2.45 nm wide device in Figure S3 in the Supplementary Materials. We conclude that the optimum range of interaction strengths exists for each device and this range is centered around −ImΣ = 2 eV for the 1c-MC configuration irrespective of PNR width, as can be seen in Figure 4a–c.

When the top contact area increases, and we consider the 3c-MC case, the findings for the 1c-MC case qualitatively hold, but the optimum −ImΣ range widens towards lower interaction strength values for all PNR widths, as shown in Figure 4d–f. Therefore, optimum −ImΣ is lower for the 3c-MC than for the 1c-MC case and equals 0.3 eV as reported earlier (see Figure 3a,b). By comparing the transmission plots of the same PNRs with different MC configurations, we conclude that increased top contact area leads to better transport properties on the low-interaction side of the plots. For *W* = 0.49 nm and at about −ImΣ ~ 0.1 eV, i.e., comparing Figure 4a,d, we observe that 3c-MCs provide higher transmission due to stronger effective broadening introduced by larger contacts. In the first case, the transmission exhibits a resonant behavior with the number of Lorentzian peaks corresponding to the number of states in the channel (Figure 4a). In contrast, the transmission is broadened and certain previously separate peaks merge into continuous high-transmission ranges when 3c-MCs are attached to the 0.49 nm wide PNR (Figure 4d). Therefore, we conclude that the optimum −ImΣ exists due to similar transmission-suppressing effects when using electrodes with either very low or very high interaction strengths. Moreover, larger top contacts provide better injection efficiency or, in other words, enable stronger effective interaction between the metal electrodes and the channel. Consequently, the range of inefficient low-interaction values becomes narrower, which in turn decreases the optimum interaction strength of the metal. As shown in Figures S4 and S5 in the Supplementary Materials, identical effects are reported for graphene nanoribbons as well in terms of DOS and transmission. Given the generality of the underlying physics, we expect our conclusions to be valid for all semiconducting 1D or quasi-1D nanostructures.

**Figure 5:**
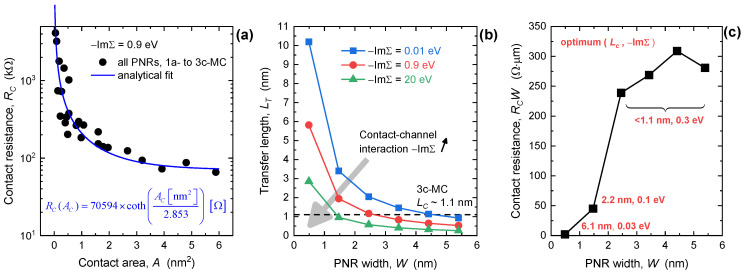
(**a**) Contact resistance vs. contact area in PNR FETs with moderately interacting MCs, i.e., −ImΣ = 0.9 eV. (**b**) Impact of PNR width downscaling on the transfer length for different metal–channel interaction strengths. (**c**) Minimum achievable added *R_C_* with corresponding optimum *L_C_* and −ImΣ.

Because the optimum electrode material changes with MC configuration, i.e., the length of the top part of the contact (*L_C_*), in the following paragraphs we investigate the transfer length (*L_T_*) for carrier injection. In Figure 5a we plot the contact area-dependent absolute *R_C_* for PNR FETs with −ImΣ = 0.9 eV. The contact area is determined by multiplying the PNR width with the length of the top part of the contact, e.g., *L_C_* = 1.1 nm for the 3c-MC case. The extracted data follows an analytical curve with a hyperbolic cotangent dependence on *A_C_* = *W*∙*L_C_*, similarly to the usual distributed resistive network model [48]. When the contact area decreases, *R_C_* is boosted significantly, which signifies poor carrier injection in PNR FETs with edge contacts and the narrowest nanoribbon channels. The characteristic area *A_T_* = *W*∙*L_T_* obtained by fitting equals 2.853 nm^2^, which enables the extraction of *L_T_*. The resulting transfer lengths are reported in Figure 5b for −ImΣ of 0.01 eV, 0.9 eV, and 20 eV, with each value representing a weakly, moderately, and strongly interacting metal. For all −ImΣ values, *L_T_* increases with the downscaling of the nanoribbon width. This behavior implies that shorter top contacts or even edge contacts are adequate only for wider PNRs and phosphorene by extension, whereas the *R_C_* of the narrowest PNRs should benefit from longer top contacts. When −ImΣ = 0.9 eV, the 3c-MC configuration provides sufficient injection area only for *W* ≥ 2.5 nm because in this width range *L_C_*~1.1 nm > *L_T_*. For narrower channels, *L_T_* increases to 5.8 nm at *W* = 0.49 nm and this demands top contacts extending over 17 unit cells (17c-MCs) for a PNR FET with the 0.49 nm wide channel. Figure 3b reports this device as well and we can see that 17c-MCs further reduce *R_C_* to 221 Ωμm at −ImΣ = 0.9 eV, i.e., by 56% when compared to the 3c-MC case that has *R_C_* of 501 Ωμm for the same interaction strength. Accordingly, the channel-size-dependent features of the transfer length show that the optimum *L_C_* exists and that it depends on PNR width and metal choice.

Altering the metal–channel interaction strength changes the *L_T_*-*W* characteristic as shown in Figure 5b. Weaker interactions (−ImΣ = 0.01 eV) result in longer transfer lengths, ranging from 0.93 nm to 10.2 nm when PNR width decreases from 5.40 nm to 0.49 nm. On the other hand, strongly interacting electrodes (−ImΣ = 20 eV) reduce *L_T_* that now varies in a smaller range between 0.26 nm to 2.85 nm for the same PNR width range. The more or less pronounced *L_T_* modulation by *W* for the weakly or strongly interacting metal, respectively, observed here for PNR FETs generally agrees with recent ab initio theoretical calculations regarding *R_C_* of metal–graphene systems [42]. Namely, it has been shown that *R_C_* of graphene devices with Ni and Ti contacts is nearly length-independent due to high −ImΣ, while the opposite is true for Pd contacts for which −ImΣ is two orders of magnitude lower than for Ni and Ti electrodes. Regarding the absolute *L_T_* values, our data provides very short transfer lengths under ~10 nm in comparison to, e.g., *L_T_* > 700 nm recorded for thick BP FET [28]. The stark difference in magnitude is due to nonideal metal–channel interfaces in the fabricated samples that include defects, tunneling and Schottky barriers, etc., that are present due to an unoptimized process or choice of electrode materials. On the other hand, optimized 2D material-based FETs such as those based on the heavily explored MoS_2_ provide *L_T_* as low as 150 nm [49], ~30 nm [50], and 5 nm [51]. Hence, our results seem to agree qualitatively and quantitatively with recent theoretical, numerical, and experimental work on metal–2D material interfaces.

Contact length in the 3c-MC configuration (~1.1 nm) is longer than *L_T_* only for PNR widths above 4.6 nm, 2.6 nm, and 1.4 nm when −ImΣ is 0.01 eV, 0.9 eV, and 20 eV, respectively. Strongly interacting metals provide superior injection and allow shorter contacts over a wider range of nanoribbon widths than other −ImΣ cases. Nevertheless, transfer length values tell us whether top contacts are long enough in terms of efficient carrier injection, and only partly inform us about the magnitude of minimum *R_C_* that is achievable in a specific device. For this reason, finding the optimum MC configuration in terms of *L_C_* and −ImΣ can be informed by data in Figure 5b, but multiple device simulations are still necessary to find minimum *R_C_* for PNR FETs with various nanoribbon widths. Figure 5c shows the *W*-dependence of minimum *R_C_* with corresponding (*L_C_*, −ImΣ) pairs needed for those *R_C_* values. We note that these values correspond to added intrinsic *R_C_* introduced by MC-induced transport effects, with tunneling and Schottky barriers assumed to be negligible. The smallest achievable contact resistance in quasi-1D phosphorene structures ranges from 281 Ωμm in wider PNRs to only 45 Ωμm in the 1.47 nm wide nanoribbon. At the same time, optimum (*L_C_*, −ImΣ) pairs change considerably with the width downscaling. The PNRs with *W* ≥ 2.45 nm prefer shorter contacts with moderately interacting metals (*L_C_* < 1.1 nm, −ImΣ = 0.3 eV), whereas the 1.47 nm wide nanoribbon benefits from longer *L_C_* and less interacting materials for contacts (*L_C_* = 2.2 nm, −ImΣ = 0.1 eV). The narrowest 0.49 nm wide PNR, which can be considered almost as a phosphorene atomic chain close in width to recently reported quasi-1D phosphorene nanostructures [18,19], exhibits the lowest *R_C_* of ~2 Ωμm. This value is reached only if *L_C_* is at least 6.1 nm and if MCs interact weakly with the channel, i.e., −ImΣ = 0.03 eV, as reported in more detail in Figures S6 and S7 in the Supplementary Materials.

Clearly, wider PNRs and large-area monolayer BP by extension prefer nearly edge contacts with moderate metal–channel interaction, which counterintuitively stands in complete contrast to the narrowest phosphorene nanoribbon devices that need long top contacts with weak coupling. Since we do not employ DFT calculations [17,52,53,54] due to the computationally prohibitive size and the number of the analyzed PNR configurations, our work does not identify exact metals for minimum *R_C_*. Moreover, additional simplifications in our approach include neglecting the impact of gate dielectrics and thermal stability [54,55,56], which could modify our findings quantitatively. Nevertheless, our study helps in identifying where to look given the previously reported data for bulk metal–phosphorene interfaces [43], and given a large amount of possibly metallic 2D material candidates with low DOS at the Fermi level and/or with weak interaction strengths [8,9].

## 4. Conclusions

Atomistic NEGF simulations of ballistic 15 nm long FETs with quasi-1D phosphorene nanostructure channels are employed to investigate the limits of contact resistance in such nanodevices. The features of *R_C_* are studied for various PNR widths in the range from ~5.5 nm down to ~0.5 nm, different hybrid edge-and-top MC configurations, and numerous metal–PNR interaction strengths. We find that optimum metals and optimum contact configurations, i.e., optimum −ImΣ and *L_C_*, exist and lead to minimum achievable MC-induced *R_C_* in phosphorene nanodevices. The presence of optimum −ImΣ is due to the separation between the two low-transmission regions, one with resonant tunneling transport when −ImΣ is small, and the other with strong broadening effects when −ImΣ is large. The optimum *L_C_* is defined by the transfer length that increases from ~1 nm to ~10 nm when the PNR width is scaled down from ~5.5 nm to ~0.5 nm. Moreover, it is shown that metals that are weakly coupled to the channel (small −ImΣ) exhibit poorer injection efficiency than those with large −ImΣ, however, this fact does not mean that weakly interacting MCs result in high *R_C_*. Surprisingly, nearly edge contacts with moderately interacting metals are preferable only for wider nanoribbons, and phosphorene by extension, whereas long top contacts with weakly interacting metals are needed for minimum *R_C_* in ultra-narrow PNRs. Specifically, for the analyzed nanoribbon width range the minimized *R_C_* ranges from 281 Ωμm (*W* = 5.40 nm, for *L_C_* < 1.1 nm and −ImΣ = 0.3 eV) to only ~2 Ωμm (*W* = 0.49 nm, for *L_C_* > 6.1 nm and −ImΣ = 0.03 eV). Our findings are supported by analyzing the underlying electronic properties and transport physics and we expect that the results should hold qualitatively for all 1D or quasi-1D nanostructures of semiconducting 2D materials. Therefore, this work could prove to be useful for minimizing *R_C_* that could enable high-performance nanodevices such as FETs, or at least facilitate the probing of relevant physical properties and phenomena that are usually obscured by the high contact resistance.

## Data Availability

The data presented in this study are contained within the article and are available on request from the corresponding author.

## Supplementary Materials

The following supporting information can be downloaded at: https://www.mdpi.com/article/10.3390/nano13111759/s1, File S1: Figure S1. Transmission of ~15 nm-long and 0.49 nm-wide PNRs with different metal contact configurations. Figure S2. Influence of contact configuration and contact-channel interaction on *R_C_* for PNR FETs with the 2.45 nm-wide nanoribbon channel. Figure S3. Transmission of 15 nm-long and 2.45 nm-wide PNRs with 1c-MCs with various −ImΣ values ranging from 0.09 eV to 20 eV. Figure S4. Impact of 1c-MCs on DOS and transmission of ~15 nm-long GNRs with different widths in the range from ~0.4 nm to ~4.8 nm. Figure S5. Impact of changing −ImΣ from 10^−2^ eV to 10^1^ eV on the transmission of ~15 nm long and ~0.4 nm-wide GNRs with different contact configurations. Figure S6. DOS and transmission of the 0.49 nm-wide PNR with 17c-MCs and metal-channel interaction parameter equal to 0.03 eV. Figure S7. Transmission of the 0.49 nm-wide PNR with 17c-MCs for different metal-channel interaction parameters between 0.03 eV and 0.1 eV.

## References

[1] Novoselov K.S., Geim A.K., Morozov S.V., Jiang D., Zhang Y., Dubonos S.V., Grigorieva I.V., Firsov A.A. (2004). Electric Field Effect in Atomically Thin Carbon Films. Science.

[2] Briggs N., Subramanian S., Lin Z., Li X., Zhang X., Zhang K., Xiao K., Geohegan D., Wallace R., Chen L.-Q. (2019). A Roadmap for Electronic Grade 2D Materials. 2D Mater..

[3] Iannaccone G., Bonaccorso F., Colombo L., Fiori G. (2018). Quantum Engineering of Transistors Based on 2D Materials Heterostructures. Nat. Nanotechnol..

[4] Zeng S., Tang Z., Liu C., Zhou P. (2021). Electronics Based on Two-Dimensional Materials: Status and Outlook. Nano Res..

[5] Vandenberghe W.G., Rostami Osanloo M. (2023). Two-Dimensional Dielectrics for Future Electronics: Hexagonal Boron Nitride, Oxyhalides, Transition-Metal Nitride Halides, and Beyond. ACS Appl. Electron. Mater..

[6] Schwierz F. (2010). Graphene Transistors. Nat. Nanotechnol..

[7] Radisavljevic B., Radenovic A., Brivio J., Giacometti V., Kis A. (2011). Single-Layer MoS2 Transistors. Nat. Nanotechnol..

[8] Haastrup S., Strange M., Pandey M., Deilmann T., Schmidt P.S., Hinsche N.F., Gjerding M.N., Torelli D., Larsen P.M., Riis-Jensen A.C. (2018). The Computational 2D Materials Database: High-Throughput Modeling and Discovery of Atomically Thin Crystals. 2D Mater..

[9] Gjerding M.N., Taghizadeh A., Rasmussen A., Ali S., Bertoldo F., Deilmann T., Knøsgaard N.R., Kruse M., Larsen A.H., Manti S. (2021). Recent Progress of the Computational 2D Materials Database (C2DB). 2D Mater..

[10] Liu H., Neal A.T., Zhu Z., Luo Z., Xu X., Tománek D., Ye P.D. (2014). Phosphorene: An Unexplored 2D Semiconductor with a High Hole Mobility. ACS Nano.

[11] Das S., Demarteau M., Roelofs A. (2014). Ambipolar Phosphorene Field Effect Transistor. ACS Nano.

[12] You H., Jia Y., Wu Z., Wang F., Huang H., Wang Y. (2018). Room-Temperature Pyro-Catalytic Hydrogen Generation of 2D Few-Layer Black Phosphorene under Cold-Hot Alternation. Nat. Commun..

[13] Miao J., Zhang L., Wang C. (2019). Black Phosphorus Electronic and Optoelectronic Devices. 2D Mater..

[14] Chaudhary V., Neugebauer P., Mounkachi O., Lahbabi S., Fatimy A.E. (2022). Phosphorene—An Emerging Two-Dimensional Material: Recent Advances in Synthesis, Functionalization, and Applications. 2D Mater..

[15] Li L., Yu Y., Ye G.J., Ge Q., Ou X., Wu H., Feng D., Chen X.H., Zhang Y. (2014). Black Phosphorus Field-Effect Transistors. Nat. Nanotechnol..

[16] Gaddemane G., Vandenberghe W.G., Van de Put M.L., Chen S., Tiwari S., Chen E., Fischetti M.V. (2018). Theoretical Studies of Electronic Transport in Monolayer and Bilayer Phosphorene: A Critical Overview. Phys. Rev. B.

[17] Szabo A., Rhyner R., Carrillo-Nunez H., Luisier M. Phonon-Limited Performance of Single-Layer, Single-Gate Black Phosphorus n- and p-Type Field-Effect Transistors. Proceedings of the IEEE International Electron Devices Meeting (IEDM).

[18] Watts M.C., Picco L., Russell-Pavier F.S., Cullen P.L., Miller T.S., Bartuś S.P., Payton O.D., Skipper N.T., Tileli V., Howard C.A. (2019). Production of Phosphorene Nanoribbons. Nature.

[19] Zhang W., Enriquez H., Tong Y., Mayne A.J., Bendounan A., Smogunov A., Dappe Y.J., Kara A., Dujardin G., Oughaddou H. (2021). Flat Epitaxial Quasi-1D Phosphorene Chains. Nat. Commun..

[20] Poljak M., Suligoj T. (2018). The Potential of Phosphorene Nanoribbons as Channel Material for Ultrascaled Transistors. IEEE Trans. Electron Devices.

[21] Poljak M., Matić M. (2022). Bandstructure and Size-Scaling Effects in the Performance of Monolayer Black Phosphorus Nanodevices. Materials.

[22] Cao X., Guo J. (2015). Simulation of Phosphorene Field-Effect Transistor at the Scaling Limit. IEEE Trans. Electron Devices.

[23] Yin D., Yoon Y. (2016). Design Strategy of Two-Dimensional Material Field-Effect Transistors: Engineering the Number of Layers in Phosphorene FETs. J. Appl. Phys..

[24] Liang G., Neophytou N., Lundstrom M.S., Nikonov D.E. (2008). Contact Effects in Graphene Nanoribbon Transistors. Nano Lett..

[25] Yoon Y., Fiori G., Hong S., Iannaccone G., Guo J. (2008). Performance Comparison of Graphene Nanoribbon FETs With Schottky Contacts and Doped Reservoirs. IEEE Trans. Electron Devices.

[26] Poljak M., Matić M. (2021). Metallization-Induced Quantum Limits of Contact Resistance in Graphene Nanoribbons with One-Dimensional Contacts. Materials.

[27] Poljak M., Matić M., Župančić T., Zeljko A. (2022). Lower Limits of Contact Resistance in Phosphorene Nanodevices with Edge Contacts. Nanomaterials.

[28] Du Y., Liu H., Deng Y., Ye P.D. (2014). Device Perspective for Black Phosphorus Field-Effect Transistors: Contact Resistance, Ambipolar Behavior, and Scaling. ACS Nano.

[29] Haratipour N., Robbins M.C., Koester S.J. (2015). Black Phosphorus P-MOSFETs with 7-Nm HfO2 Gate Dielectric and Low Contact Resistance. IEEE Electron Dev. Lett..

[30] Telesio F., le Gal G., Serrano-Ruiz M., Prescimone F., Toffanin S., Peruzzini M., Heun S. (2020). Ohmic Contact Engineering in Few–Layer Black Phosphorus: Approaching the Quantum Limit. Nanotechnology.

[31] Jena D., Banerjee K., Xing G.H. (2014). Intimate Contacts. Nat. Mater..

[32] IEEE More Moore Section International Roadmap for Devices and Systems (IRDS), 2022 Edition. https://irds.ieee.org/.

[33] Poljak M., Song E.B., Wang M., Suligoj T., Wang K.L. (2012). Influence of Edge Defects, Vacancies, and Potential Fluctuations on Transport Properties of Extremely Scaled Graphene Nanoribbons. IEEE Trans. Electron Devices.

[34] Poljak M. (2020). Electron Mobility in Defective Nanoribbons of Monoelemental 2D Materials. IEEE Electron Dev. Lett..

[35] Poljak M., Matić M., Zeljko A. (2021). Minimum Contact Resistance in Monoelemental 2D Material Nanodevices With Edge-Contacts. IEEE Electron Dev. Lett..

[36] Rudenko A.N., Katsnelson M.I. (2014). Quasiparticle Band Structure and Tight-Binding Model for Single- and Bilayer Black Phosphorus. Phys. Rev. B.

[37] Hu C. (2010). Modern Semiconductor Devices for Integrated Circuits.

[38] Sancho M.P.L., Sancho J.M.L., Sancho J.M.L., Rubio J. (1985). Highly Convergent Schemes for the Calculation of Bulk and Surface Green Functions. J. Phys. F Met. Phys..

[39] Pourfath M. (2014). The Non-Equilibrium Green’s Function Method for Nanoscale Device Simulation.

[40] Ryndyk D.A. (2016). Theory of Quantum Transport at Nanoscale: An Introduction.

[41] Nemec N., Tománek D., Cuniberti G. (2008). Modeling Extended Contacts for Nanotube and Graphene Devices. Phys. Rev. B.

[42] Fediai A., Ryndyk D.A., Cuniberti G. (2016). The Modular Approach Enables a Fully Ab Initio Simulation of the Contacts between 3D and 2D Materials. J. Phys. Condens. Matter.

[43] Pan Y., Wang Y., Ye M., Quhe R., Zhong H., Song Z., Peng X., Yu D., Yang J., Shi J. (2016). Monolayer Phosphorene–Metal Contacts. Chem. Mater..

[44] Zhang X., Pan Y., Ye M., Quhe R., Wang Y., Guo Y., Zhang H., Dan Y., Song Z., Li J. (2018). Three-Layer Phosphorene-Metal Interfaces. Nano Res..

[45] Zhong K., Xu G., Yang Y., Zhang J.-M., Huang Z. (2021). One- and Two-Dimensional Electrical Contacts and Transport Properties in Monolayer Black Phosphorene–Ni Interface. J. Phys. Condens. Matter.

[46] Fediai A., Ryndyk D.A., Seifert G., Mothes S., Claus M., Schröter M., Cuniberti G. (2016). Towards an Optimal Contact Metal for CNTFETs. Nanoscale.

[47] Li J., Sun X., Xu C., Zhang X., Pan Y., Ye M., Song Z., Quhe R., Wang Y., Zhang H. (2018). Electrical Contacts in Monolayer Blue Phosphorene Devices. Nano Res..

[48] Mitta S.B., Choi M.S., Nipane A., Ali F., Kim C., Teherani J.T., Hone J., Yoo W.J. (2020). Electrical Characterization of 2D Materials-Based Field-Effect Transistors. 2D Mater..

[49] Szabó Á., Jain A., Parzefall M., Novotny L., Luisier M. (2019). Electron Transport through Metal/MoS2 Interfaces: Edge- or Area-Dependent Process?. Nano Lett..

[50] English C.D., Shine G., Dorgan V.E., Saraswat K.C., Pop E. (2016). Improved Contacts to MoS2 Transistors by Ultra-High Vacuum Metal Deposition. Nano Lett..

[51] Cheng Z., Backman J., Zhang H., Abuzaid H., Li G., Yu Y., Cao L., Davydov A.V., Luisier M., Richter C.A. (2023). Distinct Contact Scaling Effects in MoS2 Transistors Revealed with Asymmetrical Contact Measurements. Adv. Mater..

[52] Afzalian A., Akhoundi E., Gaddemane G., Duflou R., Houssa M. (2021). Advanced DFT–NEGF Transport Techniques for Novel 2-D Material and Device Exploration Including HfS2/WSe2 van Der Waals Heterojunction TFET and WTe2/WS2 Metal/Semiconductor Contact. IEEE Trans. Electron Devices.

[53] Matić M., Poljak M. (2022). Ab Initio Quantum Transport Simulations of Monolayer GeS Nanoribbons. Solid-State Electron..

[54] Alves Machado Filho M., Hsiao C.-L., dos Santos R.B., Hultman L., Birch J., Gueorguiev G.K. (2023). Self-Induced Core–Shell InAlN Nanorods: Formation and Stability Unraveled by Ab Initio Simulations. ACS Nanosci. Au.

[55] Song S.M., Cho B.J. (2010). Investigation of Interaction between Graphene and Dielectrics. Nanotechnology.

[56] Brahma M., Van de Put M.L., Chen E., Fischetti M.V., Vandenberghe W.G. (2023). The Importance of the Image Forces and Dielectric Environment in Modeling Contacts to Two-Dimensional Materials. NPJ 2D Mater. Appl..

