# Supplementary material for: Optimum Contact Configurations for Quasi-One-Dimensional Phosphorene Nanodevices
Source: Nanomaterials (Basel). 2023 May 29;13(11):1759. doi: 10.3390/nano13111759 (PMC10254546; doi:10.3390/nano13111759)
Supplement: Supplementary file 1 [file nanomaterials-13-01759-s001.zip › nanomaterials-2425432-supplementary.pdf]

nanomaterials

Supplementary Materials

Optimum contact configurations for quasi-one-dimensional phosphorene nanodevices

Mirko Poljak* and Mislav Matić

Computational Nanoelectronics Group, Faculty of Electrical Engineering and Computing, University of Zagreb, HR 10000 Zagreb, Croatia
* Correspondence: mirko.poljak@fer.hr

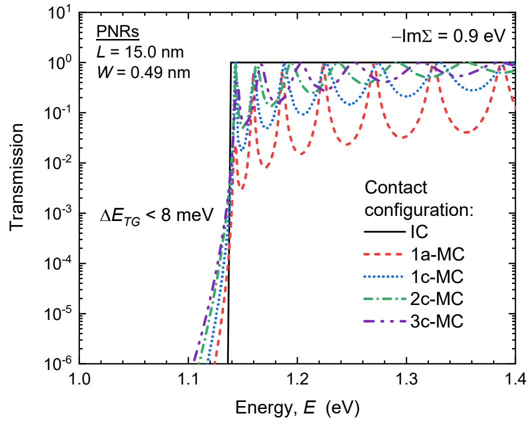

Figure S1. Transmission of ~15 nm-long and 0.49 nm-wide PNRs with different metal contact configurations. In the narrowest PNRs, only a very weak transport gap decrease (ΔE_{TG} < 8 meV) is observed even for the longest top contacts considered (3c-MC case). Device with edge contacts (1a-MC) suffers from the strongest transmission decrease near the conduction band minimum.

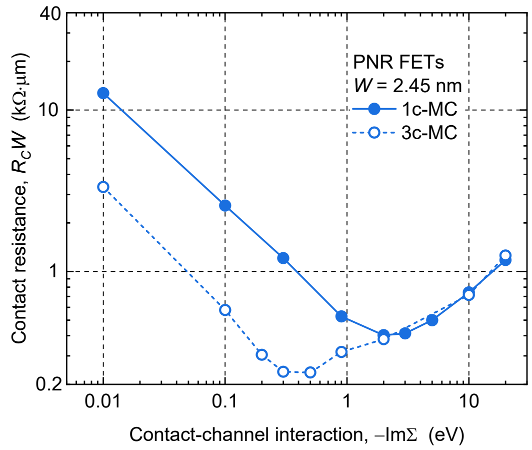

Figure S2. Influence of contact configuration (1c-MC vs. 3c-MC) and contact-channel interaction (−ImΣ) on R_C for PNR FETs with the 2.45 nm-wide nanoribbon channel. These characteristics agree with those reported in the main text for other PNR widths, indicating a common optimum −ImΣ for all quasi-1D phosphorene structures.

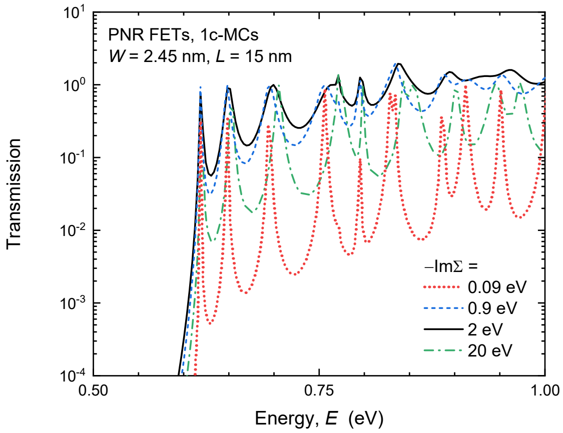

Figure S3. Transmission of 15 nm-long and 2.45 nm-wide PNRs with 1c-MCs with various −ImΣ values ranging from 0.09 eV to 20 eV. For moderate self-energy values (i.e. ~1 eV to 2 eV) the transmission curves are very similar, while transmission probability significantly deteriorates when −ImΣ either increases or decreases.

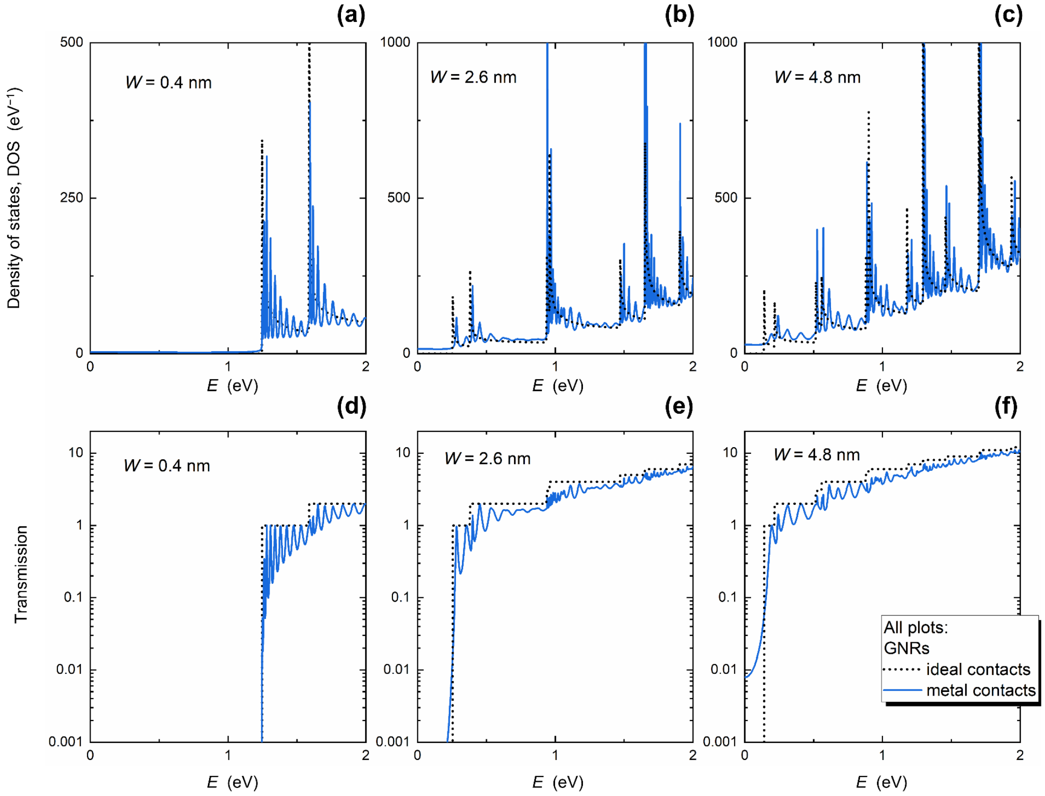

Figure S4. Impact of 1c-MCs on (a)-(c) DOS and (d)-(f) transmission of ~15 nm-long GNRs with different widths in the range from ~0.4 nm to ~4.8 nm. Metal contacts introduce Lorentzian oscillations into DOS and transmission, and MIGS inside the bandgap that increase as nanoribbon width increases. In contrast to PNRs, transport gap in wider GNRs is not preserved because the transmission inside the bandgap increases due to MC-induced broadening.

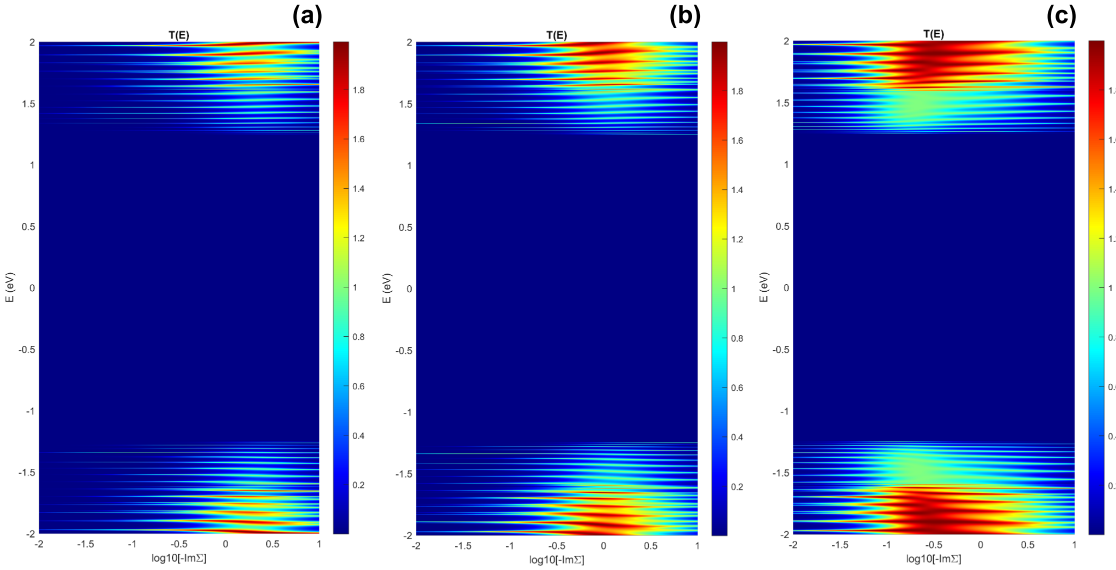

Figure S5. Impact of changing −ImΣ from 10^{-2} eV to10^{1} eV on the transmission of ~15 nm long and ~0.4 nm-wide GNRs with the following contact configurations: (a) 1a-MC or edge contacts, (b) 1c-MC, and (c) 3c-MC. When the top contact length increases, optimum interaction parameter −ImΣ moves towards lower values, exactly as in the case of PNRs, which indicates the generality of conclusions listed in the main text about optimum metal contact configurations for 1D and quasi-1D nanostructures of 2D materials.

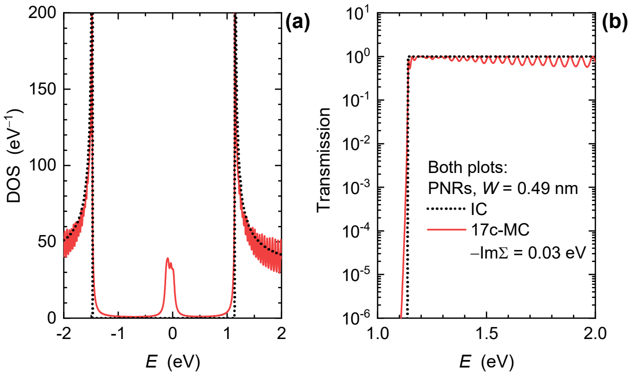

Figure S6. (a) DOS and (b) transmission of the 0.49 nm-wide PNR with 17c-MCs and metal-channel interaction parameter equal to 0.03 eV. The MIGS are induced inside the bandgap, as shown in (a), but these states are clearly localized and do not contribute to transport, as visible from the exceptionally low transmission probability inside the bandgap in (b). The transmission is high near the conduction band minimum, which benefits transport across the PNR and results in low R_C, as discussed in the main text.

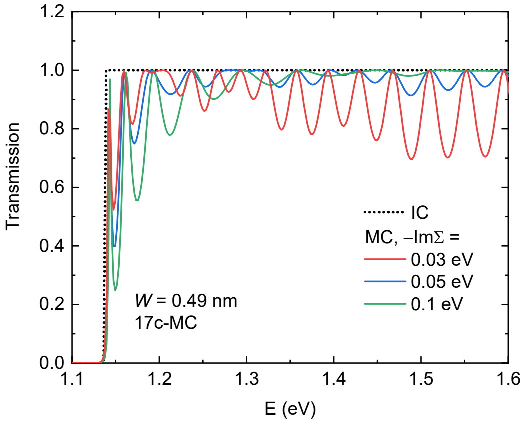

Figure S7. Transmission of the 0.49 nm-wide PNR with 17c-MCs for different metal-channel interaction parameters between 0.03 eV and 0.1 eV. Increasing −ImΣ improves the transmission only at high energies, far away from the conduction band minimum, while it decreases the transmission near the minimum, where it negatively impacts the overall device performance and R_C the most.
